# Diphtheria

**Published:** 1907-07-20

**Authors:** 


### Pathology in General Practice.

### "DIPHTHERIA."

### General Technique.

The general practitioner is constantly required to diagnose true diphtheria from inflamed or ulcerated throats, due to streptococci and other organisms; this is practically impossible without a proper bacteriological examination. The latter is usually conducted in a laboratory, the practitioner furnished with his outfit taking the swabs and forwarding them to an institution for diagnosis. But there is really no very definite reason why the individual should not carry out the necessary technique himself, if he has a small room fitted up at home as a laboratory and a small incubator included in his apparatus there. A small wooden box suitably made to contain two test tubes may be used, one of these containing a copper or iron wire with a little cotton-wool (non-antiseptic) wrapped round its end, the other solidified blood serum or Löffler's medium. These are both plugged with cotton-wool in the usual manner and sterilised. More commonly only the test tube with the wire is sent out, and this dispenses with the necessity of a box, the test tube being carried in the waistcoat pocket. When a suspected throat, nose, or larynx is to be examined, the practitioner removes the sterile wire and gently rubs the cotton-wool at its end on the membrane or ulcerated surface. He then removes the plug of the serum tube, strokes the cotton lightly on the medium, taking care not to abrade the surface, replaces the plug, and then either incubates the tube himself or sends it to a laboratory. In the second method, where no serum is supplied, the tube with the copper wire is sent to a laboratory after the swab has been rubbed on the throat, and the pathologist inoculates the medium himself, incubating it for 12 to 24 hours at 37° C. In place of the swab an ordinary looped platinum needle may be used; it is sterilised in the flame of a bunsen burner, stroked on the throat and then stroked on the medium in three parallel lines. If a distinct membrane is present a portion of this should be snipped off, dropped into the tube, and sent with the swab. On special media the diphtheria bacillus, also known as the Klebs-Löffler bacillus, grows rapidly, often appearing before any other organisms that may happen to be present have shown themselves. The colonies are whitish in colour, and appear as small circular discs, many of them separate from the line of inoculation. The appearance is often so typical as to make one at once suspect diphtheria, but though this may be so, the next part of the technique must always be followed out rigidly—that is, the microscopic examination, after suitable staining, of the colonies.

### Description of the Bacilli.

The bacilli are slender rods, straight or slightly curved, usually about 3 $\mu$ long, and a little broader than the tubercle bacillus. The size varies, however, larger and smaller varieties being found. A peculiar dotted or beaded appearance may be seen after staining, and in some instances the ends come out swollen and more deeply stained, amounting even to clubbing. They never form chains.

### Staining.

The bacillus stains well with any of the ordinary basic aniline dyes, especially methylene blue in watery solution; they retain Gram's stain, and Löffler's methylene blue is also very useful. For differential study it is usual to apply a special stain known by the name of Neisser's stain. This consists of two parts:—

(*a*) Methylene blue (Grubler), 1 gram (15 grains) is dissolved in 20 c.c. of 96-per-cent. alcohol, and to this are added 950 c.c. of distilled water and 50 c.c. of glacial acetic acid.

(*b*) Bismarck brown (Vesuvin) 2 grams, dissolved in 1 litre of distilled water.

Stain films in (*a*) 1 to 3 seconds or a little longer; wash in water; then (*b*) 3 to 5 seconds, dry and mount.

Another method of staining, which on the whole, perhaps, gives better results, is the following:—

(*a*) One minute, no washing.

(*b*) Half a minute. Wash, dry, and mount.

If the stain is satisfactory and a good result obtained, the protoplasm of the bacilli should be a faint brown colour, the granules blue, cocci and other bacilli being brown alone and showing no granules. Some make films from the membrane direct, staining them in a similar manner, but the results are never so good, and one should never give a diagnosis without making and examining a culture.

### Different Forms of the Bacilli.

Westbrook classifies the diphtheria bacilli into three groups as follows:—(1) With deeply-staining granules "granular form"; (2) with transverse bands "barred forms"; (3) those staining evenly "solid forms." He questions if this latter group be true diphtheria bacilli.

### Results to be Deduced from Finding the Diphtheria Bacillus.

In some instances what appears to be true Klebs-Löffler bacilli are found in throats that present no definite lesions. Further, there is what is termed a pseudo-diphtheria bacillus, known by the name of Hofmann's bacillus, and about this there has been considerable controversy, some believing that this is an attenuated diphtheria bacillus, others that it has no connection with diphtheria. In order to make absolutely certain with which we are dealing, further tests must be applied. The Klebs-Löffler organism produces acid in glucose broth, and "intoxicates" guinea pigs; the Hofmann does not. The last test, of course, can only be done under an experimental license, and is, fortunately, not often needed. Whenever the specific bacillus is found the practitioner notifies the disease, and the case is removed to a fever hospital, where it is generally re-examined, and if the diagnosis is confirmed, suitably treated.

